# Supplementary material for: A Cell-Penetrating Peptide Modified Cu2−xSe/Au Nanohybrid with Enhanced Efficacy for Combined Radio-Photothermal Therapy
Source: Molecules. 2023 Jan 3;28(1):423. doi: 10.3390/molecules28010423 (PMC9823383; doi:10.3390/molecules28010423)
Supplement: Supplementary file 1 [file molecules-28-00423-s001.zip › molecules-2075853-supplementary.pdf]

Supporting Information

A cell-penetrating peptide modified $Cu_{2-x}Se/Au$ nanohybrid with enhanced efficacy for combined radio-photothermal therapy

Ruixue Ran[1,#], Sinan Guo[1,#], Xiaoyu Jiang[1], Zhanyin Qian[1], Zhaoyang Guo[1], Yinsong Wang[1], Mingxin Cao[2,*], Xiaoying Yang[1,*]

[1] School of Pharmacy, Tianjin Key Laboratory on Technologies Enabling Development of Clinical Therapeutics and Diagnostics (Theranostics), School of Pharmacy, Tianjin Medical University, Tianjin, 300070, China; ranruixue@tmu.edu.cn (R.R.); 15735178933@163.com (S.G.); jiangxiaoyu115228@163.com (X.J.); qianzhanyin@tmu.edu.cn (Z.Q.); guozhaoyang@tmu.edu.cn (Z.G.); wangyinsong@tmu.edu.cn (Y.W.); yangxiaoying@tmu.edu.cn (X.W.)

[2] School and Hospital of Stomatology, Tianjin Medical University, Tianjin, 300070, China; mingxincao@tmu.edu.cn (M.C.)

* Correspondence: mingxincao@tmu.edu.cn (M.C.); yangxiaoying@tmu.edu.cn (X.Y.)

[#] These two authors equally contributed to this work.

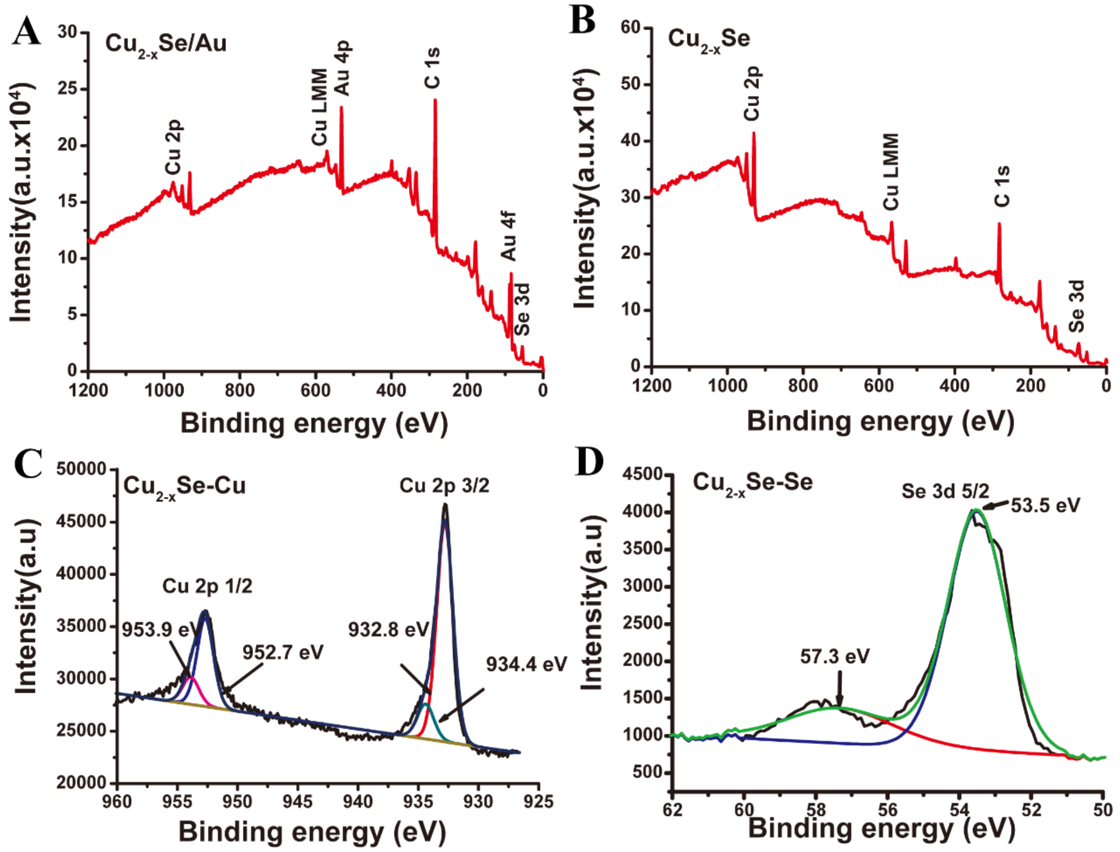

Figure S1. XPS survey spectra of (**A**) $Cu_{2-x}Se$/Au nanoparticles and (**B**) Cu_{2-x}Senanoparticles; (**C**) Cu and (**D**) Se spectra of $Cu_{2-x}Se$ nanoparticles.

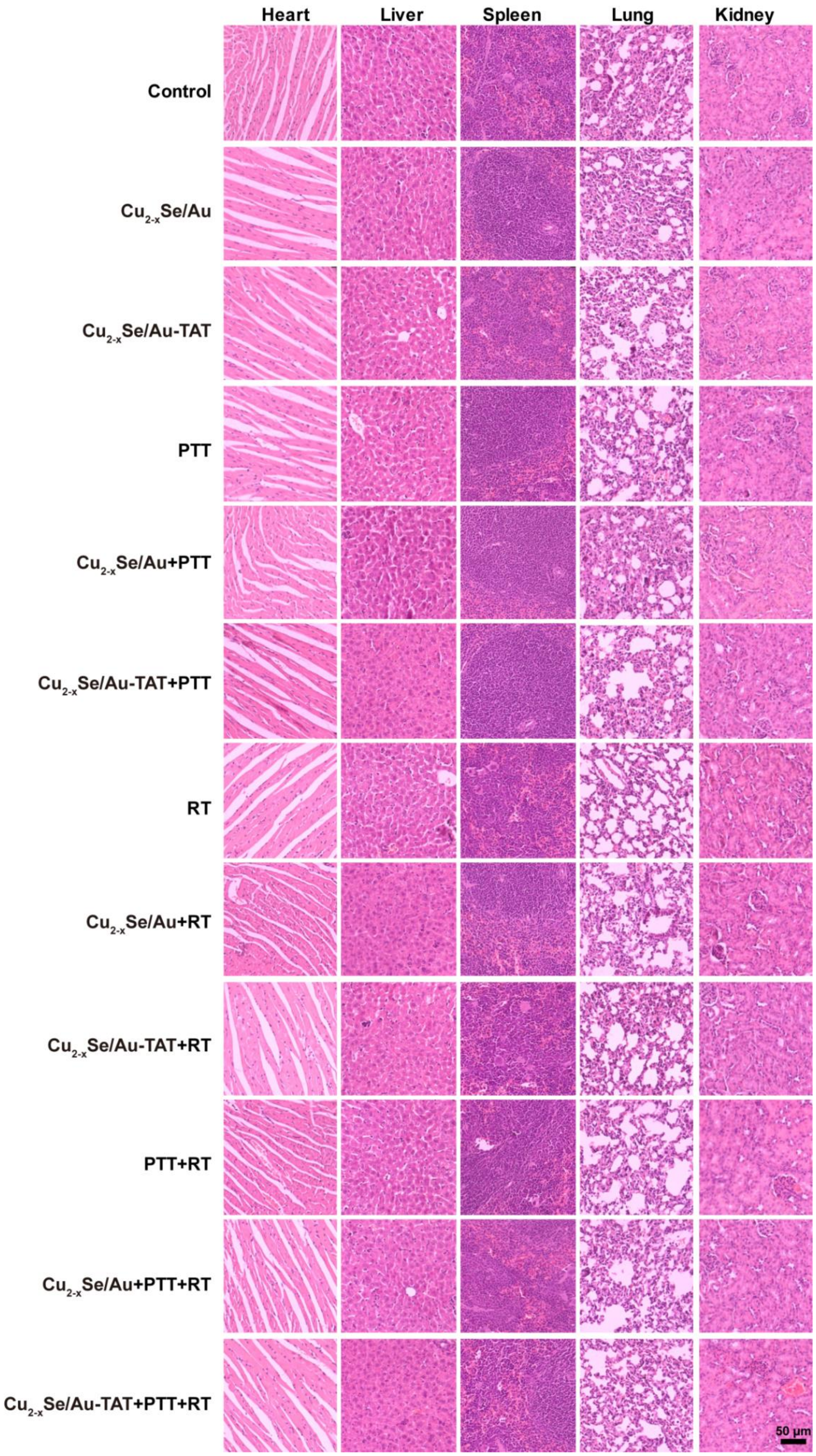

Figure S2. H&E staining images of tissue sections from the mice after different treatments.
